# Basal cell carcinoma of the prostate with squamous metaplasia: A case report and literature review

**DOI:** 10.3389/fonc.2023.1094943

**Published:** 2023-03-09

**Authors:** Junwei Wang, Cunming Zhang, Baijun Chen, Qingqing Wu

**Affiliations:** ^1^ Department of Urology, Wenling Hospital Affiliated to Wenzhou Medical University (The First People’s Hospital of Wenling), Taizhou, Zhejiang, China; ^2^ Department of Pathology, Wenling Hospital Affiliated to Wenzhou Medical University (The First People’s Hospital of Wenling), Taizhou, Zhejiang, China

**Keywords:** basal cell carcinoma, prostate, squamous metaplasia, dyspareunia, focal squamous differentiation

## Abstract

Basal cell carcinoma of the prostate (BCCP) is a rare tumor with a total incidence of 140 cases to date. However, BCCP with squamous metaplasia has not been reported as of date. In this paper, we report the first case of BCCP with squamous metaplasia. The patient was hospitalized for progressive dyspareunia and had been treated for recurrent urinary retention four times in 5 years. Rectal examination showed that the prostate was medium in texture with no palpable nodules. The levels of total prostate specific antigen (tPSA), free prostate specific antigen (fPSA), and fPSA/tPSA (f/t) ratio were 1.29 ng/mL, 0.4 ng/mL, and 0.31, respectively. Ultrasound of the urinary tract showed that the prostate gland was 51 mm*40 mm*38 mm in size. We performed transurethral resection of the prostate. Histopathology confirmed the diagnosis of basal cell carcinoma with focal squamous differentiation, and immunohistochemical staining was positive for P63 and 34βE12. A laparoscopic radical prostatectomy was performed 45 days after the first surgery and the postoperative pathology showed a small amount of residual tumor with negative margins and no involvement of the seminal vesicles and vas deferens. The patient was followed up for 50 months and was doing well by the end of our study. We describe the clinical symptoms, pathological features, treatment, and prognosis of patients with BCCP with squamous metaplasia. The relevant published literature is also briefly reviewed.

## 1 Introduction

Globally, prostate cancer is the second most common cancer in men (1). The 5- year survival rate of prostate cancer in China is 66.4%, making it the sixth most common cancer among men and the tenth most common cause of cancer death in this population (2). Prostate adenocarcinoma is the most common type of prostate cancer which originates from secretory epithelial cells (3). Basal cell carcinoma of the prostate (BCCP) is a very rare form of cancer. Based on the histological characteristics, BCCP can be divided into the adenoid cystic type and basal cell-like type, and these two different morphological manifestations can appear simultaneously (4, 5). BCCP was previously thought to be insidious but is now considered to be highly aggressive, with the potential for local spread and metastasis (6). Limited by its rarity, there is no standardized treatment for BCCP as of yet.

We report a case of BCCP presenting with difficulty in urination and hence was treated with transurethral resection of the prostate. However, the postoperative histopathology report confirmed the diagnosis of BCCP with focal squamous epithelial metaplasia. To our knowledge, this is the first case of BCCP with squamous metaplasia to be reported.

## 2 Case report

**Figure 1:**
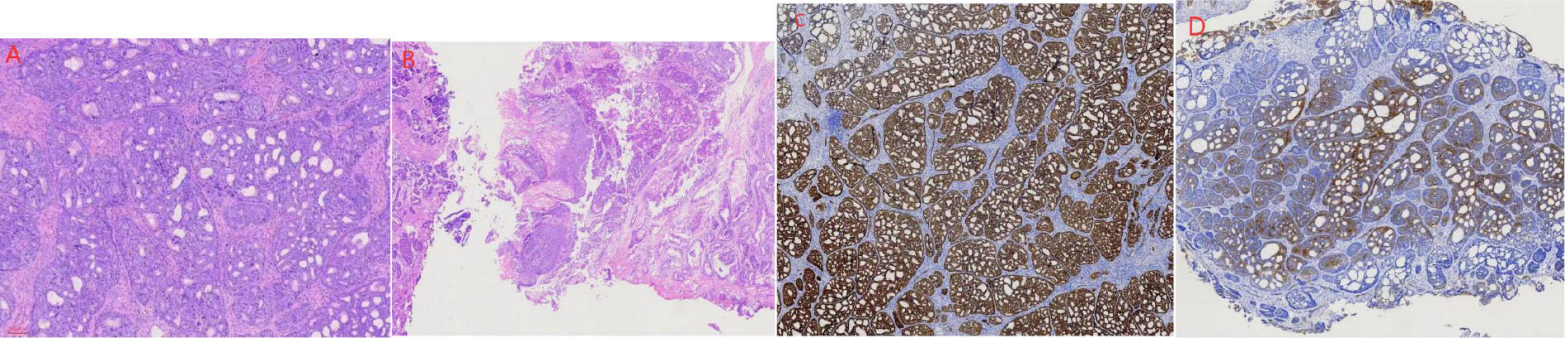
Microscopic findings of the prostate specimen. **(A)** Basal cell carcinoma of the prostate identified based on predominant adenoid cystic carcinoma morphology; **(B)** Squamous metaplasia; **(C)** Specimen section showing positive P63 marker; **(D)** Section showing positive 34 βE12 marker.

**Figure 2:**
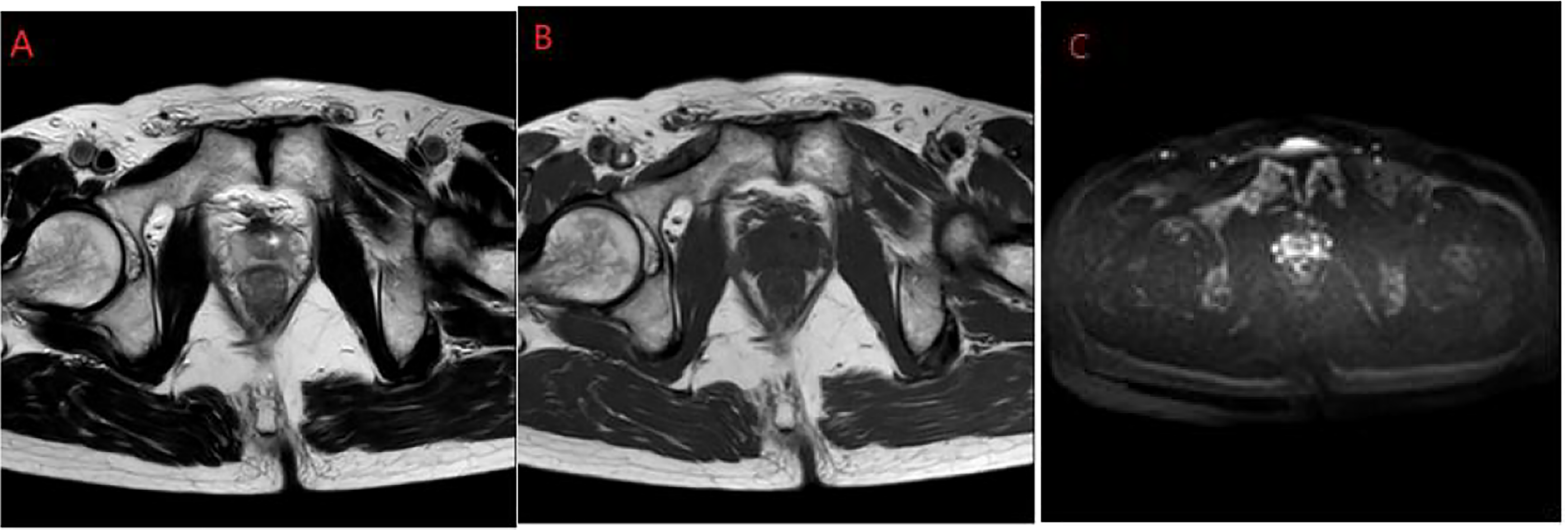
Prostate MRI. **(A)** Slightly longer T2; **(B)** Slightly longer T1; **(C)** Slightly high signal on DWI. MRI, magnetic resonance imaging; DWI, diffusion-weighted imaging.

A 59-year-old Han Chinese male patient was hospitalized for progressive dyspareunia. The patient had a history of four episodes of recurrent acute urinary retention over a 5-year period. Rectal examination revealed a smooth surface of the prostate with no palpable nodules and a medium texture. Ultrasound examination showed that the prostate was approximately 51 mm*40 mm*38 mm in size. The abdominal and lung computed tomography (CT) also reported no abnormalities. Hence, the patient was initially diagnosed with prostatic hyperplasia and was managed with transurethral resection of the prostate. However, the postoperative histopathological findings diagnosed the case as basal cell carcinoma with focal squamous differentiation (**Figures 1A, B**). Immunohistochemical staining was negative for P504s and NKX3.1 but positive for P63 (**Figure 1C**) and 34βE12 (**Figure 1D**). Microscopic examination revealed a predominantly adenoid cystic carcinoma structure with a basal cell mass of variable size containing numerous small round or ovoid window pores in the form of sieve pores with eosinophilic material. Magnetic resonance imaging (MRI) of the prostate performed 40 days postoperatively showed a small lamellar long T2 (**Figure 2A**) and long T1 signal (**Figure 2B**) in the central lobe of the prostate with a slightly high signal on diffusion-weighted imaging (DWI) (**Figure 2C**). Meanwhile, there was no difference in the morphology or signal of the seminal vesicles, and there were no abnormalities in the pelvic lymph nodes. After thorough communication with the patient, a laparoscopic radical prostatectomy was performed 45 days after the transurethral resection of the prostate. Given the patient’s young age and in accordance with their wishes, we preserved the neurovascular bundle during the operation. The postoperative histopathological examination showed a small amount ofresidual cancer with negative margins and no involvement of the seminal vesicles and vas deferens. Immunohistochemical staining revealed negative staining for P504s and NKX3.1, but positive staining for P63 and 34βE12. The patient was regularly followed up after the radical prostatectomy for up to 50 months with a final telephone follow-up on 1 October 2022. The patient reported experiencing urinary incontinence and erectile dysfunction as a result of the operation. However, his urinary incontinence gradually recovered within 3 months postoperatively, while his erectile function improved within 8 months. Prostate-specific antigen (PSA) levels checked during the follow-up period were at 0 ng/mL. No abnormalities were observed on the abdominal and lung CT images and pelvic MRI, with no signs of local recurrence or distant metastases. The patient is now well and is working normally.

## 3 Discussion

BCCP is a very rare form of cancer that accounts for 0.01% of prostate cancers (7). The first case of BCCP was reported in 1974, and the total cumulativenumber of reported cases till date is about 140 (8, 9). BCCP is usually considered to be insidious, but there are many reported cases of recurrence and metastasis (4, 10). About 44% and 10% of patients experience recurrence and death due to metastasis, respectively (5). After long-term follow-up, it was found five years (10). BCCP can be divided into two types: adenoid cystic and basal cell-like, according to the 2016 World Health Organization classification (11). However, little remains to be known of BCCP due to its rarity.

Based on the current knowledge on BCCP, its age of onset is between 28 to 97 years old, with the highest prevalence in 60 to 75 years age group (6). Approximately 89% of patients with BCCP initially present with predominantly obstructive lower urinary tract symptoms, while others present with hematuria (8). The serum PSA levels are usually within normal limits, which results in its misdiagnosis as prostatic hyperplasia (12). Its diagnosis is often confirmed by histopathological examination after transurethral resection of the prostate (8). It is commonly reported that, grossly, BCCP is yellow in color with a hard consistency (6). Further, BCCP is classified into adenoid cystic type and basal cell-like type based on histological characteristics. The basal cell-like type is characterized by large, pleomorphic nuclei with little cytoplasm, with the tumor cells arranged in a fenestrated or nested pattern; meanwhile, the adenoid cystic type is characterized by cystic dilated alveoli and sieve glands, with eosinophilic hyaline material and basophilic mucoid secretions in the lumen (4, 5).

Immunohistochemical analysis differentiates BCCP from adenocarcinoma and benign basal cell hyperplasia. P63 and 34βE12 markers are highly specific and sensitive for basal cell carcinoma of the prostate, and the diagnostic value is improved when these tests are performed together (13). Other markers such as CD44 and P53 may also be useful in the diagnosis of prostate basal cell carcinoma, but the results are inconsistent (8). The strong expression of Bcl-2 and increased Ki-67, which are associated with the grade of malignant tumors, help to distinguish it from benign basal cell hyperplasia (8, 12). There is typically no PSA or PAP immunostaining in BCCP (8).

As clinical samples of BCCP are rare, few basic studies have been performed as of date. Recently, Low et al. performed the first whole-genome sequencing of formalin- and paraffin-embedded samples from two BCCP patients and found a significant loss of chromosome 16 copy number in both cases and an overall low number of single nucleotide variants (14). In addition, whole-genome sequencing revealed several protein-coding mutations, including KIT, DENND3, PTPRU, and ITGA2, while *in vitro* validation of prostate basal cells showed a significant increase in cell proliferation in the absence of CYLD expression (14).

Due to the rarity of BCCP, no standard treatment protocol has yet been developed. Radical prostatectomy is considered to be the most effective treatment, and early radical prostatectomy can achieve good outcomes (4). Positive cut margins and peripheral nerve infiltration are indications for postoperative adjuvant radiotherapy (6). In patients with advanced BCCP, a combination of treatment modalities is often used but their efficacy is still unclear (6, 8). In contrast to prostate adenocarcinoma, basal cell carcinoma, a non-androgen-dependent tumor, has little or no androgen receptor (AR) expression, rendering endocrine therapy ineffective (4, 6). However, treatment poside was reported to be potentially effective (10). Targeted therapy against the fibroblast growth factor receptor (FGFR) has also been attempted with limited success (8).

Squamous metaplasia of the prostate originates from the basal cells of the prostatic urothelium, prostatic ductal epithelium, or prostatic alveolar epithelium (15). Furthermore, squamous metaplasia of the prostate occurs after radiotherapy or hormonal therapy in adenocarcinoma (16). However, there is no information available on the treatment and prognosis of BCCP associated with squamous metaplasia to date.

## 4 Conclusion

In conclusion, BCCP is a rare malignancy of the prostate, and we report the first case of basal cell carcinoma with squamous metaplasia. Our case adds a useful piece of puzzle that will be interesting to researchers in urological oncology field, given that the PSA levels of the patient were normal, and the final diagnosis was identified incidentally after transurethral resection of the prostate. Although the biology and prognosis of the disease are not fully understood, early treatment is the key to ensure a good prognosis.

## Data availability statement

The original contributions presented in the study are included in the article/supplementary material. Further inquiries can be directed to the corresponding author.

## Ethics statement

The studies involving human participants were reviewed and approved by Ethics Committee of Wenling First People’s Hospital. The patients/participants provided their written informed consent to participate in this study. Written informed consent was obtained from the individual(s) for the publication of any potentially identifiable images or data included in this article.

## Author contributions

Manuscript writing: JW; Clinical case diagnosis and treatment: JW, CZ, BC; Data collection and literature research: JW, CZ, BC, QW; Manuscript review and revision: CZ. All authors contributed to the article and approved the submitted version. 
